# Next-Generation Sequencing of Carbapenem-Resistant *Klebsiella pneumoniae* Strains Isolated from Patients Hospitalized in the University Hospital Facilities

**DOI:** 10.3390/antibiotics11111538

**Published:** 2022-11-03

**Authors:** Ján Koreň, Michal Andrezál, Hana Drahovská, Zuzana Hubenáková, Adriána Liptáková, Tibor Maliar

**Affiliations:** 1 Institute of Microbiology, Faculty of Medicine, Comenius University, University Hospital Bratislava, 81108 Bratislava, Slovakia; 2 Department of Molecular Biology, Faculty of Natural Sciences, Comenius University, 84215 Bratislava, Slovakia; 3 Department of Biotechnologies, Faculty of Natural Sciences, University of SS. Cyril and Methodius in Trnava, 91701 Trnava, Slovakia

**Keywords:** carbapenem-resistant *Klebsiella pneumoniae*, cgMLST, antimicrobial resistance, healthcare facilities

## Abstract

Carbapenem-resistant (CR) *Klebsiella pneumoniae* represents an urgent worldwide threat. We focused on CR *K. pneumoniae* in selected facilities of the University Hospital Bratislava (UHB) to investigate sequence types (STs), clonal relatedness, and antimicrobial resistance. Suspected carbapenem-nonsusceptible *K. pneumoniae* strains were obtained from hospitalized patients. Further examination included carbapenemase confirmation, cgMLST, and quantitative susceptibility testing. Of the total 41 CR *K. pneumoniae* strains, 26 (63.4%) were determined as ST11 in hospital No. 1; of these ST11, 22 (84.6%) were found in the first internal clinic. Six (14.6%) ST258 and three (7.3%) ST584 occurred in hospital No. 2; the most, i.e., four (66.7%), ST258 were detected in the geriatric clinic. In hospital No. 3, we found two (4.8%) ST584 and one (2.4%) ST258. Of the ST11 set, 24 (92.3%) produced NDM-1. Furthermore, seven (87.5) ST258 and five (100%) ST584 strains generated KPC-2. Antimicrobial resistance was as follows: ertapenem 97.6%, meropenem 63.4%, tigecycline 7.3%, eravacycline 7.3%, and colistin 2.5%. We revealed a presumably epidemiological association of ST11 with transmission, particularly in the first internal clinic of hospital No. 1, while ST258 and ST584 were related to interhospital dissemination between medical facilities No. 2 and No. 3. It is essential to prevent the circulation of these pathogens within and between healthcare facilities.

## 1. Introduction

Carbapenem-resistant *Enterobacteriaceae* (CRE), which include mainly *Klebsiella pneumoniae* strains, represent a significant and constantly growing problem in healthcare facilities. This concern should be considered a state-wide priority due to the increase in difficult-to-treat diseases and the high mortality rate, which is estimated to be between 33% and 42% [1]. These multidrug-resistant (MDR, i.e., resistance to three or more classes of antimicrobials) pathogens evolve primarily in a hospital setting after the exposure of patients to antibiotics, resulting in selection pressure [2,3]. Consequently, the development of resistance is dependent on the epidemiological status of the corresponding medical environment, the consumption of carbapenems, and the cross-transmission of MDR strains between patients. On the other hand, the rapid dissemination of resistance is exceedingly facilitated by β-lactamase (*bla*) genes through transferrable genetic elements [4]. Notably, resistance to carbapenems may be often associated with the presence of genes related to other antimicrobial classes; therefore, CR strains frequently appear as extensively drug-resistant (XDR, i.e., remain susceptible to only one or two antimicrobial categories) microorganisms [5]. Resistance to carbapenems linked to carbapenemase production (CP) is of greatest concern from an epidemiological point of view because carbapenemase genes located on plasmids are readily spread to many other bacterial agents [6]. The acquisition of carbapenem resistance is also possible via non-CP enzymes, such as ESBLs (extended-spectrum β-lactamases) and AmpC cephalosporinases, combined with other resistance mechanisms (porin mutations or loss, production of efflux pumps, or alteration of penicillin-binding proteins). Accordingly, the CP of CR strains must be determined and differentiated from that caused by ESBL and/or AmpC hyperproduction supported by structural mutations [4,5]. The most common carbapenemases are KPC (*Klebsiella pneumoniae* carbapenemase) from molecular class A and NDM (New Delhi metallo-β-lactamase) from class B [7], geographically related to the Slovak region and the surrounding area [4].

Regarding the global development and spread of carbapenem resistance, particularly that of *Klebsiella pneumoniae* strains, diverse national genetic and/or sequence type variations have been recorded worldwide. Using multilocus sequence typing (MLST), CR *K. pneumoniae* strains can often be associated with emergence in the relevant regional area [1]. Molecular methods enabling the detection of bacterial pathogens and their transmission have become an important tool in the control of nosocomial infections when providing care in healthcare facilities, as well as for the identification of the infection source, including the dissemination routes of epidemiological cases in the medical environment [8].

Our objective in this study was to determine the emergence of CR *Klebsiella pneumoniae* strains, their genotypic characteristics, the participation of sequence types (STs), and their genetic relatedness in connection with probable epidemiological transfer in three selected medical facilities of the University Hospital Bratislava (UHB). An important goal of this work was to investigate genes encoding antimicrobial resistance and determine the resistance of the evaluated strains to the tested antimicrobial agents.

## 2. Results

### 2.1. Isolation of CR K. pneumoniae Strains

In general, 41 CR *Klebsiella pneumoniae* strains were investigated due to presumed epidemiological transmission between hospitalized patients within three healthcare facilities belonging to the UHB. In the study cohort, there were 24 (58.5%) female patients. Patients over the age of 60 represented 90.2% (37) of the group, with a mean age of 74.7 (range of 25 to 96) years, as indicated in Table 1. In the healthcare-related setting No. 1 with 28 (68.3%) patients, the majority (23; 56.1%) originated from the first internal clinic. In the medical facility No. 2, there were ten (24.4%) individuals; of them, five (12.2%) were from the geriatric clinic. Finally, three (7.3%) CR *K. pneumoniae* strains were obtained from the Institute of Pathological Anatomy and the postmortem of patients who were treated and died in the third healthcare-associated environment within UHB. Regarding the carriage in the gastrointestinal tract, the 23 specimens were obtained from a rectal swab or stool (56.1%). As for skin and soft tissue infections, five (12.2%) samples were obtained from a wound (twice), abscess (once), or organ swab (twice). Finally, eight (19.5%) urine samples (six from infection, two from colonization) and five (12.2%) specimens from the respiratory tract (four throat swabs and one sputum sample) were obtained, as shown in Table 1. The patient conditions were designated according to previously defined criteria, including microbiological findings, as 29 (70.7%) colonizations and 12 (29.3%) infections (Supplementary Materials: Table S1a–c).

### 2.2. Genome Sequencing of CR K. pneumoniae Strains

Forty-one *Klebsiella* genomes were sequenced using the Illumina technology. We obtained high-quality whole genome contigs for all strains; the length of the genomes ranged from 5.23 to 5.66 Mbp with an average coverage of 20 to 590 times.

The clonality of the strains was determined using MLST, which revealed the presence of five sequence types (STs; Table 2). The majority of the strains belonged to the sublineage SL258, covering the ST11 (twenty-six), ST258 (eight), and ST340 (one) strains (Table 2). Five strains were assigned to ST584 of SL2004. The ST15 profile was observed once in the set.

**Figure 1:**
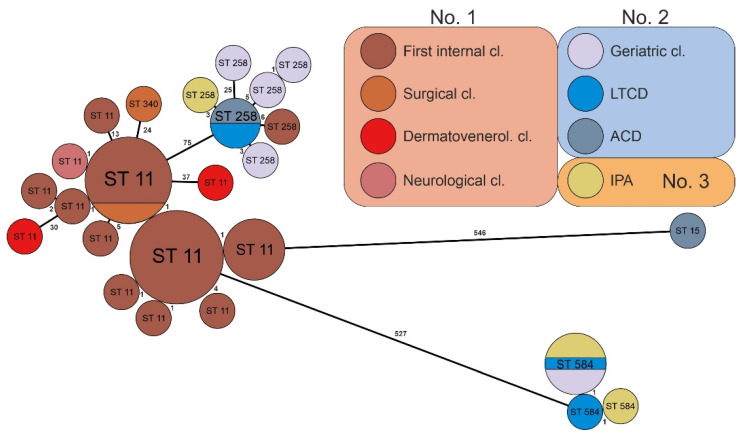
GrapeTree of 41 isolated CR *Klebsiella pneumoniae* strains based on cgMLST. Ring sizes correspond to the number of strains with the same genotype; the sequence types (STs) of the strains are marked inside the ring. The numbers on lines between rings correspond to the numbers of different alleles between groups. The color code depicts the origin of the strains—clinics, departments, or institutes, as well as the medical setting, labelled with the hospital number (**No. 1**, **No. 2**, or **No. 3)**—including clonal relatedness. **Abbreviations: cl.**—clinic; **Dermatovenerol. cl.**—dermatovenerological clinic; **LTCD**—long-term care department; **ACD**—aftercare department; **IPA**—Institute of Pathological Anatomy (strains isolated from patients who were treated and died in hospital No. 3).

The mutual relatedness of strains assigned to the same ST was further assessed using core genome MLST (cgMLST). We observed that strains belonging to the same ST differed in 0–37 cgMLST alleles (Figure 1). Based on this analysis, the ST11 strains formed thirteen unique profiles, the majority of which differed from the main group by less than ten alleles. There were three strains (KMB-944, KMB-966, and KMB-953) in which the exceptions possessed 13–37 different alleles. Similar relations were observed in ST258, where only one strain (KMB-946) showed more than ten different alleles. Five ST584 strains were found to be very closely related (Figure 1).

### 2.3. Occurrence of ST in Hospital Facilities

We observed that some *Klebsiella* sequence types occurred predominantly in some hospital settings. Twenty-two of the twenty-six ST11 strains were isolated from the first internal clinic of medical facility No. 1, and the remaining four strains were detected in other clinics of the same hospital. The ST258 and ST584 strains were predominantly isolated from healthcare facility No. 2 and partially from medical facility No. 3 (Figure 1).

The tree was constructed in GrapeTree, and the numbers above/beside the lines show the counts of different alleles.

### 2.4. Antibiotic Resistance Genes of CR K. pneumoniae Strains

The presence of antibiotic resistance genes was analyzed using the PATRIC database. All strains contained at least three genes (Table 3). Strains belonging to ST11 showed a wide spectrum of β-lactamase genes. A notable finding regarding carbapenemase activity was that 24 (92.3%) of the 26 ST11 strains produced metallo-β-lactamase NDM-1, 26 (100%) produced SHV-11, and 23 (88.4%) produced CTX-M-15, while 21 (80.7%) also produced BSBL (broad-spectrum β-lactamase) OXA-1. On the other hand, the production of the KPC-2-encoded serine carbapenemase, which was recorded in seven of the eight ST258 strains (87.5%) and in the five ST584 CR *K. pneumoniae* strains (100%), should be highlighted. TEM-1 was present in five ST258 (63%) and five ST584 (100%) strains. Seven out of the eight ST258 strains (87.5%) were positive for SHV-12 genes, and all ST584 strains contained SHV-168, CTX-M-15, and OXA-1 β-lactamase genes (Table 3).

The resistance of *K. pneumoniae* strains to antimicrobial agents was determined as follows: 100% were resistant to piperacillin/tazobactam and ceftazidime, 58.5% to ceftazidime/avibactam, 97.6% to ertapenem, 63.4% to meropenem, 78.1% to amikacin, 82.9% to gentamicin, 7.3% to tigecycline, 7.3% to eravacycline, and 2.5% to colistin, as listed in Table 4.

## 3. Discussion

CR *K. pneumoniae* strains account for a worldwide public health emergency and are designated as one of the major causes of hospital-acquired infection [9]. This is especially the case within the hospital; however, the interhospital spread of these worrisome pathogens is a very frequent and significant facilitator of the emergence of co-resistance to several antimicrobial classes [10].

We investigated 41 strains of CR *K. pneumoniae* from three UHB medical facilities and found a correlation between ST clonal similarity within these healthcare-associated settings. The mean age of the patients in our study (Table 1) was similar to that in the survey by Han et al. (2017) related to long-term acute care hospitals in the United States, where 25% of the *K. pneumoniae* clinical strains were CR *K. pneumoniae*; however, national surveillance and improved connections with acute care hospitals were decisive for reducing the spread of these pathogens [11]. The specimens used in this American observational study were from the respiratory system, urinary tract, and blood, slightly different from our specimens (Table 1); in addition, the authors used a notably larger sample size. An international multicenter cohort study [1] involving strains of CR *K. pneumoniae* isolated from patients (41% female) with a mean age of approximately 12 years or younger reported the opposite proportion to that in our research (Table 1). The authors of this intercontinental survey analyzed a large number of isolates with partially similar sample sources, including bloodstream infection, which we did not record. This research reported that most of the CR *K. pneumoniae* strains came from hospital transfers in China (64%), from long-term chronic care in the USA (28%), and even from the domestic environment in Australia (83%) and South America (74%) [1]. This diversity may be related to the variability associated with a particular region. Australian researchers [12] presented results on CR *Klebsiella* spp. causing bloodstream infections (BSIs) mainly detected in endemic areas, including Greece, Italy, and Israel. BSIs are commonly found in neutropenic and cancer patients, often requiring broad-spectrum antibiotics. This is a crucial factor for the colonization by ESBL and CR bacteria, which constitutes a reservoir for subsequent infection [12]. There were fewer patients with oncological disease in our investigated health facilities, which could be the reason why we did not obtain positive blood samples.

We found five sequence types; among these, ST11, ST15, ST258, and ST340 are widespread CR *K. pneumoniae* clones, while ST584 is rare [13]. Most strains (Table 2) were associated with ST11 in our investigation, which is in concordance with Chinese surveys of 27 provinces from 2016 to 2020 where ST11 was the main sequence type, accounting for 64.2% of the samples [14]. In our study, this clone was represented within CR *K. pneumoniae* strains at a slightly lower rate in hospital facility No. 1. High clonal relatedness was found in the first internal clinic in twelve (29.3%) and the next six (14.6%) strains (Figure 1), suggesting possible intrahospital cross-transmission within this clinic composed of three departments (A, B, and C). Other sporadic ST11 strains designated as KMB-965 from the surgical clinic and KMB-938 from the neurological clinic, were closely related to clones from the first internal clinic; this may indicate the potential dissemination of CR *K. pneumoniae* strains between the clinics in the same medical workplace—hospital No. 1 (Figure 1). The remaining ST11 strains, consisting of KMB-966, KMB-967, KMB-971, and KMB-960 from the first internal clinic, as well as KMB-944 and KMB-953 from the dermatovenerological clinic, were clonally different (Figure 1) and had less of a chance for nosocomial spreading. Raro et al. (2020) presented an investigation of carbapenemase-producing *K. pneumoniae* strains in transplanted patients and observed four epidemic clones: ST11, ST15, ST16, and ST437. ST11 was represented in 62.5% [9] of the patients, which is in line with our data. Additionally, in an Italian study of isolated CR *K. pneumoniae* strains in long-term care facilities, the sequence types ST307, ST512, and ST37 were prevalent; however, other lineages were also found (ST11, ST16, and ST253) [10]. In the long-term care department within medical facility No. 2, we found ST258 and ST584, while no ST11 was recorded. Consequently, the occurrence of sequence types can influence the transfer and variability of patients at a time and site, in a country or region, or even in a particular medical facility. CRE have achieved the remarkable endemicity and spreading of CR *K. pneumoniae*–producing KPC since 2010; the clonal group ST258 represented the majority of strains in Italy in one study [15], which is consistent with healthcare setting No. 2 in our study. Of the seven ST258 strains that were slightly mutually related, five were from hospital No. 2, including three from the geriatric clinic—KMB-942, KMB-963, and KMB-962—one from the aftercare department—KMB-940—and one from the long-term care department—KMB-948. This close clonal relatedness of the same sequence type (ST258) may indicate *intrahospital* cross-transmission of CR *K. pneumoniae* strains. Two ST258 clones designated as KMB-935, originating from hospital No. 3, and KMB-936, from the first internal clinic of hospital No. 1, were closely related to the ST258 clone lineage within hospital No. 2, which could suggest a conceivable *interhospital* transfer of the same sequence type. Furthermore, five highly related ST584 strains included KMB-968, KMB-954, and KMB-955 from healthcare facility No. 2 and KMB-969 and KMB-970 from hospital No. 3, which could illustrate the presumed dissemination of the assayed pathogens between the two medical facilities. ST584 is rare globally, while ST258 has frequently been described in the United States in previous years as the clonal expansion of dominant *K. pneumoniae* carbapenemase (KPC)-producing strains [1,11] and in Australia [16]. This *K. pneumoniae* ST258 clone is characterized by high plasticity, facilitating genetic variability [17].

The emergence of CR *K. pneumoniae* epidemics in distinct parts of the world varies along with the relation of carbapenemase and other resistance genes to sequence types [1]. Our results regarding the emergence of detected carbapenemases highlight the importance of the spread of these strains in our healthcare facilities, which may be related to the epidemiology of the respective STs as well as particular strains that generally produce carbapenemase in Slovakia and the central European region. In our examination, we determined a high association between ST11 and NDM-1 metallo-carbapenemase, including the ESBL enzymes SHV-11 and CTX-M-15. In a similar manner, a highly prevalent ST11 clone was detected in previous years in Italy, very frequently co-harboring NDM and other carbapenemases (OXA-48, VIM, and/or KPC), including ESBL, AmpC β-lactamase, aminoglycoside-modifying enzymes, fluoroquinolone resistance enzymes, and determinants of trimethoprim/sulfamethoxazole nonsusceptibility [18]. However, our results did not show the co-production of more than one carbapenemase per strain. Moreover, we noted that ST258 was more connected with KPC-2 and KPC-3 as well as SHV-12, while ST584 was completely bound to KPC-2, SHV-168, and CTX-M-15 (Table 3). In a Brazilian survey, ST11, ST16, and ST437 *K. pneumoniae* epidemic clones encompassed KPC-2 production, while ST15 created NDM-1, including carriership and a significant range of acquired resistance genes [9]. These authors reported that only 3.7% of their isolates were KPC-2 carbapenemase-producing *K. pneumoniae* ST258; however, in our results, these sequence types produced KPC-2 at a high rate (87.5%). Compared to our values, in the research from the USA, ST258 CR *K. pneumoniae* strains contained less *bla*_KPC-2_ (44%), and an even lower rate of this gene (39%) was reported in some countries of South America [1]. On the contrary, in China, ST11 was the main carrier of *bla*_KPC-2_ (94%) in the elderly [1,19] but of other carbapenemase genes (*bla*_NDM-1_) in neonates [20]. The latter corresponds to our data. Furthermore, in contrast to our results and the findings from the United States in correlation with ST258, authors from Panama published findings about NDM-producing ST258 CR *K. pneumoniae* strains (33.3%) isolated from the secretions and blood cultures of hospitalized patients [17]. The expansion of CR *K. pneumoniae* in various regions and countries has revealed more differences than similarities with regard to carbapenemase genes and additional genes encoding enzymes and/or mechanisms responsible for antimicrobial resistance against aminoglycosides, fluoroquinolones, tetracyclines, trimethoprim/sulfamethoxazole, fosfomycin, and others. The listed genetic determinants are transmitted by these clones and can be represented at a more diverse rate [9,10,21] than in our findings.

In a study by Ippolito et al. (2021), CRE, including CR *K. pneumoniae* strains, were isolated from patients with COVID-19 and ventilator-associated pneumonia (3%); however, in all Gram-negative bacteria, carbapenem resistance was achieved in up to 32% [22]. The dissemination of carbapenem resistance was facilitated by the SARS-CoV-2 pandemic; therefore, we were faced more often with the transmission of CR *K. pneumoniae* strains due to precautions focusing mainly on COVID-19 patients. Some studies reported that the prevalence of CR *K. pneumoniae* in COVID-19 patients ranged from 0.35 to 53%, and *bla*_KPC_, *bla*_OXA-48_, and *bla*_NDM_ were determined to be among the predominant genes [23].

A study published by Yin et al. (2018) on antimicrobial resistance showed that resistance against ertapenem (100%) and meropenem (95.7%) was higher, especially for meropenem (greater than 32%), when compared to our values (Table 4). In this investigation, the authors mentioned that most of these CR *K. pneumoniae* strains were NDM-producing ST278; due to their homogeneity, the carbapenem resistance profile was high [20]. Regarding the observation from China, resistance to cephalosporins—cefoperazone/sulbactam, cefuroxime, cefotaxime, ceftazidime, and cefepime—was 100%, which is in agreement with our data. Nonetheless, it should be noted that nonsusceptibility to amikacin (8.5%), gentamicin (21.3%), and ciprofloxacin (21.3%) was several times lower [20] than that in our results.

With regard to another study on ST258 CR *K. pneumoniae* strains from selected long-term acute care hospitals in 16 countries in the USA, the rate of resistance to ciprofloxacin (98.1%) is in agreement with our profile and lower against amikacin (59.2%) and tigecycline (0.7%); however, it was considerably higher against gentamicin (97.8%) and colistin (16.1%) [11]. We can comment that the results reported by the American investigators referred to ST258, which we also found, and the resistance to colistin in our data was much lower. To our knowledge, resistance could result from the administration of colistin in general treatment; however, the horizontal transfer of genes related to colistin resistance (e.g., mediated by the colistin resistance gene *mcr-1*) via plasmids can also have an impact [24]. Another study mainly analyzed the resistance of CR *K. pneumoniae* NDM producers; compared to our results, nonsusceptibility to colistin (9.8%) and tigecycline (15.9%) was higher, while it was 100% for ceftazidime/avibactam [25], which is basically intended for strains producing KPC and OXA-48. In our outcomes, resistance to eravacycline was the same as resistance to tigecycline; however, susceptibility was improved (Table 4). This synthetic fluorocycline antibacterial agent was approved for treating serious intra-abdominal infections and has an antibacterial potential that is four times greater than that of tigecycline, with a preferable concentration in serum and tissue; however, it is not indicated for the treatment of complicated urinary tract infections. Eravacycline is efficient in vitro against CRE, including strains producing KPC, NDM, VIM, IMP, and OXA-48; however, the clinical data for treatment are insufficient [26,27].

While we could rely on the improved efficacy of other more current antibacterial agents [28,29] or maybe some natural substances [30] and their derivatives that are expected in our healthcare facilities, we cannot conclude that the resistance of CR *K. pneumoniae* strains will not increase over time.

Further research is necessary to analyze genotypic determinants and detect the development of antimicrobial resistance against these dangerous MDR pathogens spreading in the medical environment as well as to find and prevent the establishment of epidemiological relationships, including clonal relatedness.

## 4. Materials and Methods

### 4.1. Hospital Setting and Patients

CR *Klebsiella pneumoniae* strains were obtained from patients residing in the University Hospital Bratislava (UHB) in the period from April 2017 to May 2019. UHB is one of the largest Slovak hospitals and consists of five healthcare workplaces. The assayed strains originated from 3 selected medical facilities, which due to anonymity preservation, were referred to as hospital No. 1, No. 2 and No. 3. The colonization of the patients was determined by a positive culture without clinical proof of infection at the time of isolation. Infection was defined on the basis of the treating physician’s diagnosis, which was compatible with the signs of clinical infection associated with the isolation of CR *K. pneumoniae* in the relevant biological sample and significant findings for microorganisms.

### 4.2. Isolation and Selection of Bacterial Strains

The identification of *Klebsiella pneumoniae* strains was carried out using the biochemical ENTEROtest 24 kit (Erba Lachema s.r.o., Czech Republic) at the Institute of Microbiology, Faculty of Medicine, Comenius University and UHB. Only one non-repetitive isolate per patient was included in the survey. The samples were collected from the various body sites of the patients and cultured on COLOREX™ ESBL screening chromogenic medium (MkB Test a.s., Rosina, Slovak Republic) and standard culture medium. Possible carbapenem resistance was predetermined by MacConkey agar with ertapenem (ERT; 10 µg) discs and Mueller–Hinton agar with ERT and meropenem (MER; 10 µg) discs. In the case of suspicious *K. pneumoniae* growth on the abovementioned screening media and/or reduced susceptibility to carbapenem antibiotics according to EUCAST criteria [31], the phenotypic colorimetric Carba NP test was carried out to prove carbapenemase production [32]. The antimicrobial susceptibility was determined using a colorimetric micromethod [33].

### 4.3. Isolation of DNA and Preparation of Libraries for NGS

Bacterial strain culture was performed on Luria–Bertani (LB) medium at 37 °C under aerobic conditions for 12 h. The Higher Purity^TM^ Bacterial Genomic DNA Isolation Kit (CanvaxBiotech; Cordoba, Spain) and the DNeasy^®^ Blood & Tissue Kit (Qiagen; Hilden, Germany) were used for DNA isolation, and DNA was quantified using the Qubit^TM^ dsDNA HS Assay Kit (Thermofisher Scientific; Eugene, OR, USA). Sequencing libraries were preprepared using the Nextera XT DNA Library Prep Kit protocol (Illumina; San Diego, CA, USA) and purified on AMPure XP magnetic beads (Beckman Coulter Life Sciences; Indianapolis, IN, USA). The quality of the library was checked using a high-sensitivity DNA electrophoresis chip and a 2100 Bioanalyzer Instrument (Agilent Technologies; Santa Clara, CA, USA). Sequencing was carried out with 2 × 150 bp reads using the Illumina NextSeq 500^TM^ platform (Illumina).

### 4.4. Bioinformatic Processing and Genome Analysis

The obtained sequencing data were assembled de novo with SPAdes using a standard setting of parameters (Center for Algorithmic Biotechnology; St. Petersburg State University, Russia) [34] and annotated using the online PATRIC program (Pathosystem Resource Integration Center) and RAST (Rapid Annotation using Subsystem Technology) software. The criterion of 100% identity and 100% coverage with the reference gene was used as the threshold for gene presence. The genomes were further analyzed using the CGE database (Center for Genomic Epidemiology; Kongens, Lyngby, Denmark) and the BIGSdb database (Institut Pasteur MLST). The standard MLST and cgMLST based on 694 genes available in the Klebsiella BIGSdb database (https://bigsdb.pasteur.fr/klebsiella/, (accessed on 6 May 2020) were used for the determination of strain relatedness [35].

GrapeTree was used for the visualization of strain clusters based on the *Klebsiella* cgMLST analysis [36]. The sequenced genomes were deposited in the *Klebsiella* MLST database (https://bigsdb.pasteur.fr/klebsiella/, (accessed on 6 May 2020) with accession numbers 12,559–12,599.

## 5. Conclusions

Although a small sample was employed in this examination, our thorough molecular analyses suggest a probable horizontal transfer with high clonal relatedness of CR *K. pneumoniae* ST11 strains in the same healthcare setting: in hospital No. 1, we observed the emergence and spread of two sequence types (ST258 and ST584) between two medical workplaces—hospitals No. 2 and No. 3—providing health care in the UHB complex. Therefore, it is enormously important to strengthen antibiotic control to prevent the development of antimicrobial resistance and to emphasize infection control and measures related to avoiding the transmission of these difficult-to-treat pathogens.

## Figures and Tables

**Table 1:** Characteristics of the patients, healthcare settings, and specimens related to 41 CR *Klebsiella pneumoniae* strains.

**Gender**		*n*	%
Male		17	41.5
Female		24	58.5
**Age (years)**			
<60		4	9.8
≥60		37	90.2
Mean (range)		74.7	(25–96)
**Setting**			
**No. 1**	First internal clinic	23	56.1
	Surgical clinic	2	4.9
	Dermatovenerological clinic	2	4.9
	Neurological clinic	1	2.4
**No. 2**	Geriatric clinic	5	12.2
	Long-term care department	3	7.3
	Aftercare department	2	4.9
**No. 3**	Institute of Pathological Anatomy	3	7.3
**Specimen**			
Rectal swab or stool		23	56.1
Wound, abscess, or organ swab		5	12.2
Urine		8	19.5
Throat swab or sputum		5	12.2

***n***—number of patients, cases, or specimens; **%**—percentage; **No. 1**, **No. 2**, and **No. 3**—designations of selected medical facilities belonging to the University Hospital Bratislava (UHB).

**Table 2:** Characterization of 41 CR *K. pneumoniae* strains.

Sublineage	Clonal Group	ST	Serotype	No of Strains
SL258	CG340	11	K15:O4	25
			K105:O2	1
		340	K15:O4	1
	CG258	258	K106:O2	7
			K107:O2	1
SL15	CG15	15	K112:O1	1
SL2004	CG584	584	K38:O3	4
			K50:O3	1

ST—sequence type; K—capsular antigen; O—specific polysaccharide antigen.

**Table 3:** Presence of antimicrobial resistance genes (%) in 41 CR *K. pneumoniae* strains according to sequence type.

*ble*_MBL_	92	0	0	0	0
*qacE*Δ*1*	92	0	75	100	0
*oqxA, oqxB20*	0	100	0	0	0
*oqxA, oqxB14*	0	0	0	0	100
*oqxA, oqxB*	100	0	100	100	0
*tet*(D)	0	0	0	100	0
*tet*(A)	11	0	0	0	100
*qnrB4*	3	0	0	0	0
*qnrB1*	0	0	0	0	100
*qnrA3*	3	0	0	0	0
*mphA*	7	0	75	0	0
*catA1/catA4*	7	0	75	0	0
*catB*	84	0	0	0	0
*fosA*	100	100	100	100	100
*Arr3*	3	0	0	0	0
*Arr2*	80	0	0	0	0
*aph(6)-Id*	3	100	0	100	100
*aph(3″)-Ib*	3	100	0	100	100
*aph(3′)-Ia*	0	0	75	0	0
*aac(6′)-Ib4*	19	0	0	0	0
*aac(6′)-Ib*	57	0	0	0	0
*aac(3)-IId*	3	0	0	100	0
*aac(3)-IIa*	76	100	0	0	100
*rmtF1*	76	0	0	0	0
*aadA16*	7	0	0	0	0
*aadA2*	88	0	75	100	0
*sul2*	3	100	0	100	100
*sul1*	92	0	75	100	0
*dfrA27*	3	0	0	0	0
*dfrA12*	88	0	75	0	0
*bla*_OXA-9_	3	0	0	0	0
*bla*_OXA-1_	80	100	0	100	100
*bla*_DHA-1_	3	0	0	0	0
*bla*_NDM-1_	92	0	0	0	0
*bla*_KPC-3_	0	0	13	0	0
*bla*_KPC-2_	0	0	87	0	100
*bla*_TEM-156_	8	0	0	0	0
*bla*_TEM-116_	4	0	0	0	0
bla_TEM-1_	11	100	63	100	100
*bla*_CTX-M-15_	88	100	0	100	100
*bla*_SHV-168_	0	0	0	0	100
*bla*_SHV-28_	0	100	0	0	0
*bla*_SHV-27_	0	0	0	0	0
*bla*_SHV-12_	0	0	87	0	0
*bla*_SHV-11_	100	0	13	100	0
	**ST11**	**ST15**	**ST258**	**ST340**	**ST584**

The listed gene-encoding products confer resistance [mechanism] against the following agents: **Ble-MBL**—bleomycin [bleomycin-binding protein]; **QacE delta 1**—quaternary ammonium compounds; **OqxA, OqxB20, OqxB14, OqxB**—[efflux pumps] providing resistance to tetracyclines, nitrofurans, diaminopyrimidines, fluoroquinolones, and glycylcyclines; **Tet(D)**, **Tet(A)**—tetracyclines; **QnrB4**, **QnrB1**, **QnrA3**—fluoroquinolone antibiotics (enoxacin, ciprofloxacin, levofloxacin, moxifloxacin); **MphA**—macrolides (erythromycin, roxithromycin, azithromycin); **CatA1/CatA4**, **CatB**—chloramphenicol [chloramphenicol O-acetyltransferase]; **FosA**—fosfomycin [fosfomycin thiol transferase]; **Arr3**, **Arr2**—rifamycin antibiotics such as rifampin; **APH(6)-Id**, **APH(3″)-Ib**—streptomycin; **APH(3′)-Ia**—kanamycin, neomycin, paromomycin; **AAC(6′)-Ib4**, **AAC(6′)-Ib**—tobramycin, amikacin; **AAC(3)-IId; AAC(3)-IIa**—tobramycin, gentamicin; **Rmtf1**—aminoglycosides (neomycin, amikacin, gentamicin); **AadA16**, **AadA2**—ANT(3″)-Ia [group of aminoglycoside nucleotidyl transferases], resistance to aminoglycosides (streptomycin, spectinomycin); **Sul2**, **Sul1**—sulfonamides; **Dfra27**, **Dfra12**—diaminopyrimidine antibiotics such as trimethoprim; **OXA-9**, **OXA-1**—betalactams (cephalosporins, penicillins); **DHA-1**—betalactams (cephalosporins, cephamycin, penicillins); **NDM-1**—betalactams (cephalosporins, carbapenems, penicillins, cephamycin); **KPC-3**, **KPC-2**—betalactams (cephalosporins, carbapenems, penicillins, cephamycin); **TEM-156**, **TEM-116**; **TEM-1**—betalactams (cephalosporins, penicillins, penems, monobactams); **CTX-M-15**, **SHV-28**, **SHV-168**, **SHV-27**, **SHV-12**, **SHV-11**—betalactams (cephalosporins, penicillins, monobactams).

**Table 4:** Antimicrobial resistance of 41 carbapenem-resistant *Klebsiella pneumoniae* strains (µg/mL).

Antibiotics	MIC Range			MIC_50_	MIC_90_	%R	%S
Cefoperazone/sulbactam	32	-	128	128	128	100	0
Piperacillin/tazobactam	64	-	128	128	128	100	0
Cefuroxime	32	-	64	64	64	100	0
Cefotaxime	16	-	64	64	64	100	0
Ceftazidime	32	-	64	64	64	100	0
Ceftazidime/avibactam	0.064	-	64	64	64	58.5	41.5
Cefepime	16	-	64	64	64	100	0
Ertapenem	0.25	-	8	8	8	97.6	2.4
Meropenem	0.125	-	32	32	32	63.4	21.9
Amikacin	0.5	-	128	128	128	78.1	21.9
Gentamicin	0.25	-	32	32	32	82.9	17.1
Tobramycin	0.5	-	32	32	32	97.6	2.4
Ciprofloxacin	2	-	8	8	8	100	0
Tigecycline	0.5	-	8	1	2	7.3	68.3
Eravacycline	0.25	-	2	0.5	0.5	7.3	90.2
Colistin	0.25	-	4	1	2	2.5	97.5
Trimethoprim/sulfamethoxazole	1	-	8	8	8	80.5	7.3

MIC—minimum inhibitory concentration; R—resistant; S—susceptible.

## Data Availability

Not applicable.

## Supplementary Materials

The following supporting information can be downloaded at: https://www.mdpi.com/article/10.3390/antibiotics11111538/s1, Table S1a, Sequencing parameters; Table S1b, Genome data; Table S1c, Antibiotic resistance.
