# Supplementary material for: Next-Generation Sequencing of Carbapenem-Resistant Klebsiella pneumoniae Strains Isolated from Patients Hospitalized in the University Hospital Facilities
Source: Antibiotics (Basel). 2022 Nov 3;11(11):1538. doi: 10.3390/antibiotics11111538 (PMC9686475; doi:10.3390/antibiotics11111538)
Supplement: Supplementary file 1 [file antibiotics-11-01538-s001.zip › Supplementary Table S1a, Sequencing parameters.pdf]

Strain	Alternative name	Year of isolation	Hospital	Department	Sequencing platform	No. of contigs	Average coverage	Total length [bp]	L50	N50 [bp]	Acc. No.
KMB-938	2894	2017	No. 1	Neurological cl.	NextSeq 500/550	86	171	5 475 068	12	167 543	12559
KMB-967	186	2019	No. 1	First internal cl.	NextSeq 500/550	120	145	5 377 389	16	123 598	12560
KMB-960	5260	2018	No. 1	First internal cl.	NextSeq 500/550	137	116	5 532 942	16	117 498	12561
KMB-931	2151	2017	No. 1	First internal cl.	NextSeq 500/550	96	172	5 506 009	12	167 543	12562
KMB-944	5702	2017	No. 1	Dermatovenerol. cl.	NextSeq 500/550	143	76	5 467 001	16	108 744	12563
KMB-932	2158	2017	No. 1	First internal cl.	NextSeq 500/550	107	170	5 520 511	14	146 121	12564
KMB-943	5301	2017	No. 1	First internal cl.	NextSeq 500/550	105	188	5 516 680	12	145 948	12565
KMB-947	110	2018	No. 1	First internal cl.	NextSeq 500/550	132	151	5 518 078	14	121 652	12566
KMB-933	2806	2017	No. 1	First internal cl.	NextSeq 500/550	92	210	5 520 774	12	178 044	12567
KMB-934	2721	2017	No. 1	First internal cl.	NextSeq 500/550	111	147	5 518 909	13	150 873	12568
KMB-945	10	2018	No. 1	First internal cl.	NextSeq 500/550	136	207	5 521 789	16	112 908	12569
KMB-971	2655	2019	No. 1	First internal cl.	NextSeq 500/550	134	183	5 520 507	15	117 509	12570
KMB-952	3626	2018	No. 1	First internal cl.	NextSeq 500/550	146	134	5 567 028	17	100 646	12571
KMB-956	4980	2018	No. 1	First internal cl.	NextSeq 500/550	137	150	5 523 990	14	122 135	12572
KMB-957	5200	2018	No. 1	First internal cl.	NextSeq 500/550	127	229	5 519 971	13	139 206	12573
KMB-958	5371	2018	No. 1	First internal cl.	NextSeq 500/550	134	196	5 524 418	13	142 995	12574
KMB-961	5496	2018	No. 1	First internal cl.	NextSeq 500/550	145	149	5 562 698	14	126 203	12575
KMB-964	4769	2018	No. 1	First internal cl.	NextSeq 500/550	134	213	5 522 480	13	144 506	12576
KMB-959	5344	2018	No. 1	First internal cl.	NextSeq 500/550	134	168	5 522 304	14	113 007	12577
KMB-937	3613	2017	No. 1	First internal cl.	NextSeq 500/550	98	593	5 535 987	11	178 02	12578
KMB-953	3878	2018	No. 1	Dermatovenerol. cl.	NextSeq 500/550	143	139	5 258 003	18	95 900	12579
KMB-965	4871	2018	No. 1	Surgical cl.	NextSeq 500/550	137	103	5 558 200	16	113 024	12580
KMB-941	4381	2017	No. 1	First internal cl.	NextSeq 500/550	98	162	5 595 043	11	172 968	12581
KMB-950	2874	2018	No. 1	First internal cl.	NextSeq 500/550	143	149	5 563 756	15	115 687	12582
KMB-951	2961	2018	No. 1	First internal cl.	NextSeq 500/550	146	191	5 564 181	15	109 956	12583
KMB-966	6272	2018	No. 1	First internal cl.	NextSeq 500/550	165	65	5 405 440	16	104 872	12584
KMB-949	1191	2018	No. 2	ACD	NextSeq 500/550	118	150	5 634 091	17	111 922	12585
KMB-942	5087	2017	No. 2	Geriatric cl.	NextSeq 500/550	106	134	5 510 799	15	116 275	12586
KMB-948	200	2018	No. 2	LTCD	NextSeq 500/550	125	171	5 452 950	16	100 646	12587
KMB-940	4949	2017	No. 2	ACD	NextSeq 500/550	122	82	5 551 061	15	113 754	12588
KMB-936	3593	2017	No. 1	First internal cl.	NextSeq 500/550	143	19	5 523 030	16	116 182	12589
KMB-946	64	2018	No. 2	Geriatric cl.	NextSeq 500/550	140	95	5 665 505	15	118 726	12590
KMB-962	5734	2018	No. 2	Geriatric cl.	NextSeq 500/550	156	134	5 533 044	15	112 785	12591
KMB-963	5678	2018	No. 2	Geriatric cl.	NextSeq 500/550	159	170	5 525 045	14	121 911	12592
KMB-935	3541	2017	No. 3	IPA	NextSeq 500/550	148	95	5 616 766	17	120 974	12593
KMB-939	4018	2017	No. 1	Surgical cl.	NextSeq 500/550	112	148	5 449 992	12	167 543	12594
KMB-954	3862	2018	No. 2	LTCD	NextSeq 500/550	130	135	5 458 308	12	125 919	12595
KMB-955	3837	2018	No. 2	Geriatric cl.	NextSeq 500/550	157	80	5 484 544	12	137 202	12596
KMB-968	1797	2019	No. 2	LTCD	NextSeq 500/550	141	66	5 420 638	11	172 862	12597
KMB-969	2435	2019	No. 3	IPA	NextSeq 500/550	169	49	5 407 025	17	92 298	12598
KMB-970	1906	2019	No. 3	IPA	NextSeq 500/550	102	132	5 256 961	9	172 705	12599
