# Supplementary material for: Next-Generation Sequencing of Carbapenem-Resistant Klebsiella pneumoniae Strains Isolated from Patients Hospitalized in the University Hospital Facilities
Source: Antibiotics (Basel). 2022 Nov 3;11(11):1538. doi: 10.3390/antibiotics11111538 (PMC9686475; doi:10.3390/antibiotics11111538)
Supplement: Supplementary file 1 [file antibiotics-11-01538-s001.zip › Supplementary Table S1b, Genome data.pdf]

Strain	Alternati-ve name	Year of isolation	Hospital	Department	Phylo-group	Sub-lineage	Clonal group	ST	Capsule	Resistance genes (encoding products)																								
										bla TEM	*bla* SHV	*bla* CTX-M	*bla* KPC	*bla* NDM	*bla* DHA	*bla* OXA	*dfra*	*sul*	*aad*	*rmtF1*	*aac(3)*	*aac(6')*	*aph(3')*	*aph(3'')*	*aph(6)*	*Arr*	*fosA*	*cat*	*mphA*	*qnr*	*tet*	*oqx*	*qacE Δ1*	*ble* MBL
KMB-938	2894	2017	No. 1	Neurological cl.	Kp1	SL258	CG340	11	K15:O4	-	SHV-11	-	-	NDM-1	-	-	-	-	-	-	-	-	-	-	-	-	FosA	-	-	-	-	OqxA, OqxB	-	Ble-MBL
KMB-967	186	2019	No. 1	First internal cl.	Kp1	SL258	CG340	11	K15:O4	-	SHV-11	-	-	NDM-1	-	-	Dfra12	Sul1	AadA2	-	-	-	-	-	-	-	FosA	-	-	-	-	OqxA, OqxB	QacE delta 1	Ble-MBL
KMB-960	5260	2018	No. 1	First internal cl.	Kp1	SL258	CG340	11	K15:O4	TEM-1	SHV-11	-	-	NDM-1	-	OXA-1	Dfra27	Sul1	AadA16	RmtF1	AAC(3)-IId	AAC(6')-Ib	-	-	-	-	FosA	CatB	mphA	QnrA3	-	OqxA, OqxB	QacE delta 1	Ble-MBL
KMB-931	2151	2017	No. 1	First internal cl.	Kp1	SL258	CG340	11	K15:O4	-	SHV-11	CTX-M-15	-	NDM-1	-	-	Dfra12	Sul1	AadA2	RmtF1	-	AAC(6')-Ib	-	-	-	Arr2	FosA	CatB	-	-	-	OqxA, OqxB	QacE delta 1	Ble-MBL
KMB-944	5702	2017	No. 1	Dermatovenerol. cl.	Kp1	SL258	CG340	11	K105:O2	TEM-1	SHV-11	CTX-M-15	-	-	DHA-1	OXA-1, OXA-9	Dfra12	Sul1	AadA2, AadA16	-	AAC(3)-IIa	-	-	-	-	-	FosA	CatA1/CatA4	-	QnrB4	-	OqxA, OqxB	-	-
KMB-932	2158	2017	No. 1	First internal cl.	Kp1	SL258	CG340	11	K15:O4	TEM-116	SHV-11	CTX-M-15	-	NDM-1	-	-	Dfra12	Sul1	AadA2	RmtF1	-	AAC(6')-Ib	-	-	-	Arr2	FosA	CatB	-	-	-	OqxA, OqxB	QacE delta 1	Ble-MBL
KMB-943	5301	2017	No. 1	First internal cl.	Kp1	SL258	CG340	11	K15:O4	-	SHV-11	CTX-M-15	-	NDM-1	-	OXA-1	Dfra12	Sul1	AadA2	-	AAC(3)-IIa	AAC(6')-Ib	-	-	-	Arr2	FosA	CatB	-	-	-	OqxA, OqxB	QacE delta 1	Ble-MBL
KMB-947	110	2018	No. 1	First internal cl.	Kp1	SL258	CG340	11	K15:O4	-	SHV-11	CTX-M-15	-	NDM-1	-	OXA-1	Dfra12	Sul1	AadA2	RmtF1	-	AAC(6')-Ib4	-	-	-	Arr2	FosA	CatB	-	-	-	OqxA, OqxB	QacE delta 1	Ble-MBL
KMB-933	2806	2017	No. 1	First internal cl.	Kp1	SL258	CG340	11	K15:O4	-	SHV-11	CTX-M-15	-	NDM-1	-	OXA-1	Dfra12	Sul1	AadA2	RmtF1	AAC(3)-IIa	AAC(6')-Ib	-	-	-	Arr2	FosA	CatB	-	-	-	OqxA, OqxB	QacE delta 1	Ble-MBL
KMB-934	2721	2017	No. 1	First internal cl.	Kp1	SL258	CG340	11	K15:O4	-	SHV-11	CTX-M-15	-	NDM-1	-	OXA-1	Dfra12	Sul1	AadA2	RmtF1	AAC(3)-IIa	AAC(6')-Ib	-	-	-	Arr2	FosA	CatB	-	-	-	OqxA, OqxB	QacE delta 1	Ble-MBL
KMB-945	10	2018	No. 1	First internal cl.	Kp1	SL258	CG340	11	K15:O4	-	SHV-11	CTX-M-15	-	NDM-1	-	OXA-1	Dfra12	Sul1	AadA2	RmtF1	AAC(3)-IIa	AAC(6')-Ib	-	-	-	Arr2	FosA	CatB	-	-	-	OqxA, OqxB	QacE delta 1	Ble-MBL
KMB-971	2655	2019	No. 1	First internal cl.	Kp1	SL258	CG340	11	K15:O4	-	SHV-11	CTX-M-15	-	NDM-1	-	OXA-1	Dfra12	Sul1	AadA2	RmtF1	AAC(3)-IIa	AAC(6')-Ib	-	-	-	Arr2	FosA	CatB	-	-	-	OqxA, OqxB	QacE delta 1	Ble-MBL
KMB-952	3626	2018	No. 1	First internal cl.	Kp1	SL258	CG340	11	K15:O4	-	SHV-11	CTX-M-15	-	NDM-1	-	OXA-1	Dfra12	Sul1	AadA2	RmtF1	AAC(3)-IIa	AAC(6')-Ib	-	-	-	Arr2	FosA	CatB	-	-	-	OqxA, OqxB	QacE delta 1	Ble-MBL
KMB-956	4980	2018	No. 1	First internal cl.	Kp1	SL258	CG340	11	K15:O4	-	SHV-11	CTX-M-15	-	NDM-1	-	OXA-1	Dfra12	Sul1	AadA2	RmtF1	AAC(3)-IIa	AAC(6')-Ib	-	-	-	Arr2	FosA	CatB	-	-	-	OqxA, OqxB	QacE delta 1	Ble-MBL
KMB-957	5200	2018	No. 1	First internal cl.	Kp1	SL258	CG340	11	K15:O4	-	SHV-11	CTX-M-15	-	NDM-1	-	OXA-1	Dfra12	Sul1	AadA2	RmtF1	AAC(3)-IIa	AAC(6')-Ib	-	-	-	Arr2	FosA	CatB	-	-	-	OqxA, OqxB	QacE delta 1	Ble-MBL
KMB-958	5371	2018	No. 1	First internal cl.	Kp1	SL258	CG340	11	K15:O4	-	SHV-11	CTX-M-15	-	NDM-1	-	OXA-1	Dfra12	Sul1	AadA2	RmtF1	AAC(3)-IIa	AAC(6')-Ib	-	-	-	Arr2	FosA	CatB	-	-	-	OqxA, OqxB	QacE delta 1	Ble-MBL
KMB-961	5496	2018	No. 1	First internal cl.	Kp1	SL258	CG340	11	K15:O4	-	SHV-11	CTX-M-15	-	NDM-1	-	OXA-1	Dfra12	Sul1	AadA2	RmtF1	AAC(3)-IIa	AAC(6')-Ib	-	-	-	Arr2	FosA	CatB	-	-	-	OqxA, OqxB	QacE delta 1	Ble-MBL
KMB-964	4769	2018	No. 1	First internal cl.	Kp1	SL258	CG340	11	K15:O4	-	SHV-11	CTX-M-15	-	NDM-1	-	OXA-1	Dfra12	Sul1	AadA2	RmtF1	AAC(3)-IIa	AAC(6')-Ib	-	-	-	Arr2	FosA	CatB	-	-	-	OqxA, OqxB	QacE delta 1	Ble-MBL
KMB-959	5344	2018	No. 1	First internal cl.	Kp1	SL258	CG340	11	K15:O4	-	SHV-11	CTX-M-15	-	NDM-1	-	OXA-1	Dfra12	Sul1	AadA2	RmtF1	AAC(3)-IIa	AAC(6')-Ib4	-	-	-	Arr2	FosA	CatB	-	-	-	OqxA, OqxB	QacE delta 1	Ble-MBL
KMB-937	3613	2017	No. 1	First internal cl.	Kp1	SL258	CG340	11	K15:O4	-	SHV-11	CTX-M-15	-	NDM-1	-	OXA-1	Dfra12	Sul1	AadA2	RmtF1	AAC(3)-IIa	AAC(6')-Ib4	-	-	-	Arr2	FosA	CatB	-	-	-	OqxA, OqxB	QacE delta 1	Ble-MBL
KMB-953	3878	2018	No. 1	Dermatovenerol. cl.	Kp1	SL258	CG340	11	K15:O4	-	SHV-11	CTX-M-15	-	NDM-1	-	OXA-1	Dfra12	Sul1	AadA2	RmtF1	AAC(3)-IIa	AAC(6')-Ib4	-	-	-	Arr2	FosA	CatB	-	-	Tet(A)	OqxA, OqxB	QacE delta 1	Ble-MBL
KMB-965	4871	2018	No. 1	Surgical cl.	Kp1	SL258	CG340	11	K15:O4	-	SHV-11	CTX-M-15	-	NDM-1	-	OXA-1	Dfra12	Sul1	AadA2	RmtF1	AAC(3)-IIa	AAC(6')-Ib	-	-	-	Arr2	FosA	CatB	-	-	Tet(A)	OqxA, OqxB	QacE delta 1	Ble-MBL
KMB-941	4381	2017	No. 1	First internal cl.	Kp1	SL258	CG340	11	K15:O4	-	SHV-11	CTX-M-15	-	NDM-1	-	OXA-1	Dfra12	Sul1	AadA2	-	AAC(3)-IIa	AAC(6')-Ib4	-	-	-	Arr2	FosA	CatB	-	-	Tet(A)	OqxA, OqxB	QacE delta 1	Ble-MBL
KMB-950	2874	2018	No. 1	First internal cl.	Kp1	SL258	CG340	11	K15:O4	TEM-156	SHV-11	CTX-M-15	-	NDM-1	-	OXA-1	Dfra12	Sul1	AadA2	RmtF1	AAC(3)-IIa	-	-	-	-	Arr2	FosA	CatB	-	-	-	OqxA, OqxB	QacE delta 1	Ble-MBL
KMB-951	2961	2018	No. 1	First internal cl.	Kp1	SL258	CG340	11	K15:O4	TEM-156	SHV-11	CTX-M-15	-	NDM-1	-	OXA-1	Dfra12	Sul1	AadA2	RmtF1	AAC(3)-IIa	-	-	-	-	Arr2	FosA	CatB	-	-	-	OqxA, OqxB	QacE delta 1	Ble-MBL
KMB-966	6272	2018	No. 1	First internal cl.	Kp1	SL258	CG340	11	K15:O4	TEM-1	SHV-11	CTX-M-15	-	-	-	OXA-1	-	Sul1, Sul2	-	-	AAC(3)-IIa	-	-	APH(3'')-Ib	APH(6)-Id	Arr3	FosA	CatA1/CatA4	mphA	-	-	OqxA, OqxB	QacE delta 1	-
KMB-949	1191	2018	No. 2	ACD	Kp1	SL15	CG15	15	K112:O1	TEM-1	SHV-28	CTX-M-15	-	-	-	OXA-1	-	Sul2	-	-	AAC(3)-IIa	-	-	APH(3'')-Ib	APH(6)-Id	-	FosA	-	-	-	-	OqxA, OqxB20	-	-
KMB-942	5087	2017	No. 2	Geriatric cl.	Kp1	SL258	CG258	258	K106:O2	TEM-1	SHV-12	-	KPC-2	-	-	-	-	-	-	-	-	-	-	-	-	-	FosA	-	-	-	-	OqxA, OqxB	-	-
KMB-948	200	2018	No. 2	LTCD	Kp1	SL258	CG258	258	K106:O2	TEM-1	SHV-12	-	KPC-2	-	-	-	-	-	-	-	-	-	-	-	-	-	FosA	-	-	-	-	OqxA, OqxB	-	-
KMB-940	4949	2017	No. 2	ACD	Kp1	SL258	CG258	258	K106:O2	-	SHV-12	-	KPC-2	-	-	-	Dfra12	Sul1	AadA2	-	-	-	APH(3')-Ia	-	-	-	FosA	CatA1/CatA4	mphA	-	-	OqxA, OqxB	QacE delta 1	-
KMB-936	3593	2017	No. 1	First internal cl.	Kp1	SL258	CG258	258	K106:O2	-	SHV-12	-	KPC-2	-	-	-	Dfra12	Sul1	AadA2	-	-	-	APH(3')-Ia	-	-	-	FosA	CatA1/CatA4	mphA	-	-	OqxA, OqxB	QacE delta 1	-
KMB-946	64	2018	No. 2	Geriatric cl.	Kp1	SL258	CG258	258	K107:O2	-	SHV-11	-	KPC-3	-	-	-	Dfra12	Sul1	AadA2	-	-	-	APH(3')-Ia	-	-	-	FosA	CatA1/CatA4	mphA	-	-	OqxA, OqxB	QacE delta 1	-
KMB-962	5734	2018	No. 2	Geriatric cl.	Kp1	SL258	CG258	258	K106:O2	TEM-1	SHV-12	-	KPC-2	-	-	-	Dfra12	Sul1	AadA2	-	-	-	APH(3')-Ia	-	-	-	FosA	CatA1/CatA4	mphA	-	-	OqxA, OqxB	QacE delta 1	-
KMB-963	5678	2018	No. 2	Geriatric cl.	Kp1	SL258	CG258	258	K106:O2	TEM-1	SHV-12	-	KPC-2	-	-	-	Dfra12	Sul1	AadA2	-	-	-	APH(3')-Ia	-	-	-	FosA	CatA1/CatA4	mphA	-	-	OqxA, OqxB	QacE delta 1	-
KMB-935	3541	2017	No. 3	IPA	Kp1	SL258	CG258	258	K106:O2	TEM-1	SHV-12	-	KPC-2	-	-	-	Dfra12	Sul1	AadA2	-	-	-	APH(3')-Ia	-	-	-	FosA	CatA1/CatA4	mphA	-	-	OqxA, OqxB	QacE delta 1	-
KMB-939	4018	2017	No. 1	Surgical cl.	Kp1	SL258	CG340	340	K15:O4	TEM-1	SHV-11	CTX-M-15	-	-	-	OXA-1	-	Sul1, Sul2	AadA2	-	AAC(3)-IId	-	-	APH(3'')-Ib	APH(6)-Id	-	FosA	-	-	-	Tet(D)	OqxA, OqxB	QacE delta 1	-
KMB-954	3862	2018	No. 2	LTCD	Kp1	SL2004	CG584	584	K38:O3	TEM-1	SHV-168	CTX-M-15	KPC-2	-	-	OXA-1	-	Sul2	-	-	AAC(3)-IIa	-	-	APH(3'')-Ib	APH(6)-Id	-	FosA	-	-	QnrB1	Tet(A)	OqxA, OqxB14	-	-
KMB-955	3837	2018	No. 2	Geriatric cl.	Kp1	SL2004	CG584	584	K38:O3	TEM-1	SHV-168	CTX-M-15	KPC-2	-	-	OXA-1	-	Sul2	-	-	AAC(3)-IIa	-	-	APH(3'')-Ib	APH(6)-Id	-	FosA	-	-	QnrB1	Tet(A)	OqxA, OqxB14	-	-
KMB-968	1797	2019	No. 2	LTCD	Kp1	SL2004	CG584	584	K38:O3	TEM-1	SHV-168	CTX-M-15	KPC-2	-	-	OXA-1	-	Sul2	-	-	AAC(3)-IIa	-	-	APH(3'')-Ib	APH(6)-Id	-	FosA	-	-	QnrB1	Tet(A)	OqxA, OqxB14	-	-
KMB-969	2435	2019	No. 3	IPA	Kp1	SL2004	CG584	584	K50:O3	TEM-1	SHV-168	CTX-M-15	KPC-2	-	-	OXA-1	-	Sul2	-	-	AAC(3)-IIa	-	-	APH(3'')-Ib	APH(6)-Id	-	FosA	-	-	QnrB1	Tet(A)	OqxA, OqxB14	-	-
KMB-970	1906	2019	No. 3	IPA	Kp1	SL2004	CG584	584	K38:O3	TEM-1	SHV-168	CTX-M-15	KPC-2	-	-	OXA-1	-	Sul2	-	-	AAC(3)-IIa	-	-	APH(3'')-Ib	APH(6)-Id	-	FosA	-	-	QnrB1	Tet(A)	OqxA, OqxB14	-	-
