# Supplementary material for: Next-Generation Sequencing of Carbapenem-Resistant Klebsiella pneumoniae Strains Isolated from Patients Hospitalized in the University Hospital Facilities
Source: Antibiotics (Basel). 2022 Nov 3;11(11):1538. doi: 10.3390/antibiotics11111538 (PMC9686475; doi:10.3390/antibiotics11111538)
Supplement: Supplementary file 1 [file antibiotics-11-01538-s001.zip › Supplementary Table S1c, Antibiotic resistance.pdf]

Strain	Alternati-ve name	Year of isolation	Hospital	Department	ST	ESBL	B/E/AmpC	KARB	amikacin	[µg/mL]	tobramycin	[µg/mL]	gentamicin	[µg/mL]	piperacillin/ tazobactam	[µg/mL]	meropenem	[µg/mL]	ertapenem	[µg/mL]	cefoperazone/ sulbactam	[µg/mL]	ceftazidime	[µg/mL]	ceftazidime/ avibactam	[µg/mL]	cefotaxime	[µg/mL]	cefuroxime	[µg/mL]	cefepime	[µg/mL]	ciprofloxacin	[µg/mL]	tigecycline	[µg/mL]	eravacycline	[µg/mL]	colistin	[µg/mL]	trimethoprim/ sulfamethoxazole	[µg/mL]
KMB-938	2894	2017	No. 1	Neurological cl.	11	–	EDTA	NDM	R	32	R	8	R	8	R	128	R	16	R	8	R	128	R	64	R	64	R	64	R	64	R	64	R	8	S	1	S	0.38	S	2	R	8
KMB-967	186	2019	No. 1	First internal cl.	11	–	EDTA	NDM	S	0.5	S	0.5	S	0.25	R	128	R	32	R	8	R	128	R	64	R	64	R	64	R	64	R	32	R	8	S	0.5	S	0.5	S	0.25	R	8
KMB-960	5260	2018	No. 1	First internal cl.	11	–	EDTA	NDM	R	128	R	32	R	32	R	128	I	8	R	8	R	128	R	64	R	64	R	64	R	64	R	32	R	8	S	1	S	0.38	S	0.5	I	4
KMB-931	2151	2017	No. 1	First internal cl.	11	ESBL	EDTA	NDM	R	128	R	32	R	32	R	128	R	32	R	8	R	128	R	64	R	64	R	64	R	64	R	64	R	8	S	1	S	0.25	S	1	R	8
KMB-944	5702	2017	No. 1	Dermatovenerol. cl.	11	ESBL	AmpC	neg.	R	64	R	32	R	32	R	128	S	0.25	R	1.5	R	128	R	64	S	0.064	R	64	R	64	R	64	R	8	S	1	S	0.38	S	2	R	8
KMB-932	2158	2017	No. 1	First internal cl.	11	ESBL	EDTA	NDM	R	128	R	32	R	32	R	128	R	16	R	8	R	128	R	64	R	64	R	64	R	64	R	64	R	8	S	0.5	S	0.25	S	1	S	2
KMB-943	5301	2017	No. 1	First internal cl.	11	ESBL	EDTA	NDM	R	128	R	32	R	32	R	128	R	32	R	8	R	128	R	64	R	64	R	64	R	64	R	64	R	8	S	1	S	0.38	S	0.25	R	8
KMB-947	110	2018	No. 1	First internal cl.	11	ESBL	EDTA	NDM	R	64	R	32	R	32	R	128	I	4	R	8	R	128	R	64	R	64	R	64	R	64	R	64	R	8	S	0.5	S	0.25	S	0.5	R	8
KMB-933	2806	2017	No. 1	First internal cl.	11	ESBL	EDTA	NDM	R	128	R	32	R	32	R	128	R	32	R	8	R	64	R	64	R	64	R	64	R	64	R	64	R	8	S	1	S	0.38	S	1	R	8
KMB-934	2721	2017	No. 1	First internal cl.	11	ESBL	EDTA	NDM	R	128	R	32	R	32	R	128	R	32	R	8	R	128	R	64	R	64	R	64	R	64	R	64	R	8	S	1	S	0.38	S	2	R	8
KMB-945	10	2018	No. 1	First internal cl.	11	ESBL	EDTA	NDM	R	128	R	32	R	32	R	128	I	8	R	8	R	128	R	64	R	64	R	64	R	64	R	64	R	8	S	0.5	S	0.25	S	0.5	R	8
KMB-971	2655	2019	No. 1	First internal cl.	11	ESBL	EDTA	NDM	R	128	R	32	R	32	R	128	R	32	R	8	R	128	R	64	R	64	R	64	R	64	R	64	R	2	S	0.5	S	0.38	S	1	I	4
KMB-952	3626	2018	No. 1	First internal cl.	11	ESBL	EDTA	NDM	R	128	R	32	R	32	R	128	R	32	R	8	R	128	R	64	R	64	R	64	R	64	R	64	R	8	R	4	S	0.25	S	2	R	8
KMB-956	4980	2018	No. 1	First internal cl.	11	ESBL	EDTA	NDM	R	128	R	32	R	32	R	128	S	2	R	8	R	128	R	64	R	64	R	64	R	64	R	64	R	8	S	0.5	S	0.38	S	0.25	I	4
KMB-957	5200	2018	No. 1	First internal cl.	11	ESBL	EDTA	NDM	R	128	R	32	R	32	R	128	R	16	R	8	R	128	R	64	R	64	R	64	R	64	R	64	R	8	S	0.5	S	0.38	S	0.25	R	8
KMB-958	5371	2018	No. 1	First internal cl.	11	ESBL	EDTA	NDM	R	128	R	32	R	32	R	128	I	4	R	8	R	128	R	64	R	64	R	64	R	64	R	64	R	8	S	1	S	0.38	S	0.5	I	4
KMB-961	5496	2018	No. 1	First internal cl.	11	ESBL	EDTA	NDM	R	128	R	32	R	32	R	64	R	32	R	8	R	128	R	64	R	64	R	64	R	64	R	64	R	8	R	4	S	0.25	R	4	R	8
KMB-964	4769	2018	No. 1	First internal cl.	11	ESBL	EDTA	NDM	R	128	R	32	R	32	R	128	I	4	R	8	R	128	R	64	R	64	R	16	R	64	R	64	R	8	S	1	S	0.38	S	0.5	S	1
KMB-959	5344	2018	No. 1	First internal cl.	11	ESBL	EDTA	NDM	R	128	R	32	R	32	R	128	R	32	R	8	R	128	R	64	R	64	R	64	R	64	R	64	R	8	I	2	S	0.5	S	0.25	R	8
KMB-937	3613	2017	No. 1	First internal cl.	11	ESBL	EDTA	NDM	R	128	R	32	R	32	R	128	R	32	R	8	R	64	R	64	R	64	R	32	R	64	R	64	R	8	S	0.5	S	0.38	S	2	R	8
KMB-953	3878	2018	No. 1	Dermatovenerol. cl.	11	ESBL	EDTA	NDM	R	128	R	32	R	32	R	128	I	8	R	8	R	128	R	64	R	64	R	64	R	64	R	64	R	8	I	2	S	0.38	S	0.5	R	8
KMB-965	4871	2018	No. 1	Surgical cl.	11	ESBL	EDTA	NDM	R	128	R	32	R	32	R	128	R	32	R	8	R	128	R	64	R	64	R	64	R	64	R	64	R	8	I	2	S	0.5	S	1	I	4
KMB-941	4381	2017	No. 1	First internal cl.	11	ESBL	EDTA	NDM	R	128	R	32	R	32	R	128	R	32	R	8	R	128	R	64	R	64	R	64	R	64	R	64	R	8	I	2	I	0.75	S	1	R	8
KMB-950	2874	2018	No. 1	First internal cl.	11	ESBL	EDTA	NDM	R	128	R	32	R	32	R	128	S	0.5	R	4	R	128	R	64	R	64	R	64	R	64	R	64	R	8	S	1	R	2	S	0.25	R	8
KMB-951	2961	2018	No. 1	First internal cl.	11	ESBL	EDTA	NDM	R	128	R	32	R	32	R	128	S	2	R	4	R	128	R	64	R	64	R	64	R	64	R	64	R	8	S	0.5	S	0.38	S	0.5	R	8
KMB-966	6272	2018	No. 1	First internal cl.	11	ESBL		neg.	S	8	R	32	R	32	R	128	R	16	R	8	R	128	R	64	S	1	R	64	R	64	R	64	R	4	S	1	S	0.38	S	0.5	R	8
KMB-949	1191	2018	No. 2	ACD	15	ESBL		neg.	S	2	R	32	R	32	R	128	S	2	R	8	R	128	R	64	S	0.125	R	64	R	64	R	64	R	8	I	2	S	0.38	S	0.25	R	8
KMB-942	5087	2017	No. 2	Geriatric cl.	258	ESBL	BA	KPC	R	128	R	32	S	1	R	128	R	16	R	8	R	128	R	64	S	1	R	64	R	32	R	64	R	8	S	0.5	S	0.5	S	1	S	1
KMB-948	200	2018	No. 2	LTCD	258	ESBL	BA	KPC	R	64	R	16	S	0.5	R	128	R	32	R	1	R	128	R	64	S	2	R	64	R	64	R	64	R	8	I	2	R	2	S	2	R	8
KMB-940	4949	2017	No. 2	ACD	258	ESBL	BA	KPC	R	32	R	32	R	32	R	128	R	32	R	8	R	128	R	64	S	0.5	R	64	R	64	R	64	R	8	S	1	S	0.5	S	2	R	8
KMB-936	3593	2017	No. 1	First internal cl.	258	ESBL	BA	KPC	R	64	R	32	S	1	R	128	R	32	R	8	R	128	R	64	S	1	R	32	R	64	R	64	R	4	S	0.5	S	0.38	S	0.25	R	8
KMB-946	64	2018	No. 2	Geriatric cl.	258	ESBL	BA	KPC	R	16	R	32	R	32	R	128	R	32	R	8	R	128	R	64	S	2	R	64	R	64	R	64	R	8	S	0.5	S	0.25	S	1	R	8
KMB-962	5734	2018	No. 2	Geriatric cl.	258	ESBL	BA	KPC	R	128	R	32	S	2	R	128	R	32	R	8	R	128	R	64	S	0.5	R	64	R	64	R	64	R	8	S	1	S	0.38	S	0.5	R	8
KMB-963	5678	2018	No. 2	Geriatric cl.	258	ESBL	BA	KPC	R	128	R	32	S	1	R	128	R	32	R	8	R	128	R	64	S	0.5	R	64	R	64	R	64	R	8	S	0.5	S	0.38	S	1	R	8
KMB-935	3541	2017	No. 3	IPA	258	ESBL	BA	KPC	R	32	R	16	S	1	R	128	R	32	R	8	R	128	R	64	S	0.5	R	64	R	64	R	64	R	8	S	0.5	R	2	S	2	R	8
KMB-939	4018	2017	No. 1	Surgical cl.	340	ESBL		neg.	S	2	R	8	R	32	R	128	S	0.125	S	0.25	R	32	R	32	S	0.5	R	64	R	64	R	32	R	8	I	2	S	0.38	S	1	R	8
KMB-954	3862	2018	No. 2	LTCD	584	ESBL	BA	KPC	S	2	R	16	R	32	R	128	S	2	R	8	R	32	R	64	S	0.5	R	64	R	64	R	16	R	4	I	2	S	0.5	S	0.25	R	8
KMB-955	3837	2018	No. 2	Geriatric cl.	584	ESBL	BA	KPC	S	4	R	32	R	32	R	128	S	2	R	8	R	32	R	64	S	0.5	R	64	R	64	R	32	R	8	R	8	S	0.5	S	0.25	R	8
KMB-968	1797	2019	No. 2	LTCD	584	ESBL	BA	KPC	S	8	R	32	R	32	R	128	R	32	R	8	R	128	R	64	S	0.5	R	64	R	64	R	64	R	8	I	2	S	0.5	S	1	R	8
KMB-969	2435	2019	No. 3	IPA	584	ESBL	BA	KPC	S	8	R	32	R	32	R	128	R	32	R	8	R	128	R	64	S	1	R	64	R	64	R	64	R	8	I	2	S	0.5	-	-	R	8
KMB-970	1906	2019	No. 3	IPA	584	ESBL	BA	KPC	S	4	R	32	R	32	R	64	S	2	R	4	R	64	R	64	S	0.5	R	16	R	64	R	64	R	8	S	0.5	S	0.5	S	0.25	R	8
